# Dissecting diffuse large B-cell lymphomas of the “not otherwise specified” type: the impact of molecular techniques

**DOI:** 10.12688/f1000research.16755.1

**Published:** 2018-12-21

**Authors:** Stefano A Pileri, Enrico Derenzini, Federica Melle, Giovanna Motta, Angelica Calleri, Pierluigi Antoniotti, Virginia Maltoni, Sebastiano Spagnolo, Stefano Fiori, Valentina Tabanelli, Marco Fabbri

**Affiliations:** 1 Haematopathology Division, European Institute of Oncology, Milan, Italy

**Keywords:** Diffuse Large B cell Lymphoma, Sequencing, Gene expression, Microenvironment

## Abstract

The updated edition of the Classification of Tumours of Haematopoietic and Lymphoid Tissues, published in September 2017 by the World Health Organization (WHO), presents many important changes to the document published in 2008. Most of these novelties are linked to the exceptional development of biomolecular techniques during the last 10 years. To illustrate how much new technologies have contributed to the better classification of single entities, as well as the discovery of new ones, would go beyond the objectives of this work. For this reason, we will take diffuse large B-cell lymphoma as an example of the cognitive improvement produced by high-yield technologies (such as the gene expression profile, the study of copy number variation, and the definition of the mutational spectrum). The acquisition of this knowledge not only has a speculative value but also represents the elements for effective application in daily practice. On the one hand, it would allow the development of personalised therapy programs, and on the other it would promote the transition from the bench of the researcher's laboratory to the patient's bedside.

## Introduction

In the revised World Health Organization (WHO) Classification of Tumours of Haematopoietic and Lymphoid Tissues, diffuse large B-cell lymphoma (DLBCL) represents the commonest type of lymphoid malignancy. Although some rare variants of the tumour are recognised, the process is usually characterised by the proliferation of large B cells (measuring ≥20 µm in diameter) and shows quite variable cytological features that hamper a more precise and reproducible subdivision, justifying the suffix “NOS” (not otherwise specified) ^1^.

For decades, the classification of DLBCL was based on morphologic criteria ^2, 3^ integrated with phenotypic attributes. However, its application showed more and more that it had no prognostic or therapeutic impact ^4, 5^. In the Revised WHO Classification of Tumours of Haematopoietic and Lymphoid Tumours, two important novelties have been introduced in the setting of DLBCL/NOS: a) the definition of the cell of origin (COO) has become mandatory, and b) the new provisional category of high-grade B-cell lymphomas, with special reference to those carrying double hits/triple hits (DH/TH), has been introduced ^1^. EBV-positive DLBCL may represent a further novelty, although it represents just an expansion of the previous category of DLBCL of the elderly, quoted in the fourth edition of the WHO Classification ^1^. In fact, it was found that EBV-positive DLBCL (excluding lymphomatoid granulomatosis, plasmablastic lymphoma, DLBCL associated with chronic inflammation, and muco-cutaneous ulcer, all entities related to EBV infection) is not confined to elderly people—as originally thought—but can present over a wide age range ^6^. Finally, yet importantly, the usage of next-generation sequencing (NGS), although not yet mandatory, is strongly encouraged, representing an important tool in the present era of precision medicine.

## Cell of origin determination

In February 2000, in a seminal paper published in Nature, Alizadeh and co-workers (National Cancer Institute [NCI] of the United States of America) first reported that DLBCLs could be subdivided in at least two major categories based on their gene signature. Such distinction was unfeasible on morphologic grounds. The two categories were termed germinal centre B cell-like (GCB) and activated B cell-like (ABC) depending on whether their gene signatures were closer to those of germinal centre cells or activated B-lymphocytes circulating in the peripheral blood, respectively. The molecular subclassification was relevant not only for histogenetic reasons but also, most importantly, for prognosis and therapy ^7^. In fact, by using the CHOP standard chemotherapy, there was a dramatic difference (of about 50 percentage points) in terms of progression-free survival (PFS) and overall survival (OS) between GCB and ABC DLBCLs, the latter having the worse clinical course. Such a difference has been maintained in the present immunochemotherapy era ^8, 9^. Two years later, the data published by the NCI group were validated by a transatlantic consortium, the Lymphoma/Leukemia Molecular Profiling Project (LLMPP), on a much larger series of cases by using a comprehensive gene chip. The LLMPP study allowed the identification of a third group that was intermediate between the GCB and ABC ones (termed “unclassified” [U]) ^9– 11^.

The main limitation of these studies was the need for profiling mRNA extracted from fresh or frozen tissue, which is available in only a small minority of patients who are referred to leading institutions. Thus, attempts were made to substitute the results of gene expression profiling (GEP) with immunohistochemistry (IHC) algorithms (Hans, Choi, Colomo, Muris, Pileri, and Tally) based on a limited number of markers and applicable to formalin-fixed, paraffin-embedded (FFPE) tissue samples ^12– 18^. However, the Lunenburg Biomarker Consortium demonstrated the extreme variability of results when the same algorithm was applied at nine institutions with extensive experience in the field of haematopathology. These discrepancies were due to the usage of different antigen retrieval techniques, detection methods, and platforms along with interpersonal and intrapersonal variability in the interpretation of the results obtained. Secondly, the comparison of the classification of DLBCL based on the COO profoundly diverged in most studies when GEP and IHC results were compared. The GelCab, for instance, reported lack of prognostic value of two algorithms (Hans and Tally) when applied to the same cases conversely to GEP. Such divergence can be explained by the fact that GEP subdivides DLBCLs into three groups (GCB, ABC, and U), while IHC divides DLBCLs into GCB and non-GCB, the latter group being a kind of waste-basket ^19, 20^. In contrast, Visco *et al*. found a 92% association between GEP and IHC in a study including 475 *de novo* DLBCLs. The former was performed on FFPE samples and in selected cases on frozen tissue by the HG-U133 Plus 2.0 Gene Chip and Affymetrix platform, while the latter was carried out by applying an IHC algorithm based on the detection of CD10, FOXP1, and BCL6 to tissue microarrays. In light of the need for optimally fixed FFPE tissue to perform conventional GEP, it would be interesting to compare the results published in 2012 ^18^ with those obtainable on the same cases by the Lymph2Cx (see below).

In 2014, the LLMPP proposed a new approach based on a 20-gene panel (15 top-genes and five housekeeping genes), known as Lymph2Cx, which reproduced the results of conventional GEP by using mRNA extracted from FFPE tissue samples. The analysis was carried out on the NanoString platform that measured the exact amount of mRNA expressed by a given gene without retrotranscription or amplification. The Lymph2Cx turned out to be superior to three IHC algorithms (Hans, Choi, and Tally) when applied to 67 DLBCLs all treated with R-CHOP and provided with both FFPE and frozen tissue available. OS and PFS curves appeared over-imposable by profiling FFPE and frozen samples on the NanoString and Affymetrix platforms, respectively ^21^. The NanoString approach produced identical results when different platforms were employed. Finally, the Lymph2Cx allowed the detection of the third group (U) of DLBCLs shown by conventional GEP ^9, 11^. Importantly, while IHC regarded 33% and 67% of the cases as GCB and non-GCB, respectively, with no differences in terms of OS and PFS, targeted GEP classified 60%, 25%, and 15% of the cases as GCB, ABC, and U by showing significantly different responses to therapy. The LLMPP results were later confirmed by independent studies (BCCA, LYSA, and GOYA) based on larger series of cases all treated with R-CHOP or R-CHOP-like therapy ^22– 24^. Two further reports published in the *British Journal of Haematology* and *Journal of Clinical Oncology* highlighted that the impact of COO determination by Lymph2Cx might show limitations under some circumstances ^25, 26^. The former suggested that the correct histogenetic classification might lose its prognostic impact in patients older than 70 because of frequent comorbidities ^25^. The latter, although confirming the lack of relationship between IHC and targeted GEP, showed that the use of aggressive immunochemotherapy regimens (R-CHOP14 in the elderly and R-MegaCHOEP21 in younger individuals) can improve the response of the ABC forms by annulling the prognostic difference among the three molecular subgroups ^26^.

In our experience, based on profiling more than 300 DLBCLs by the Lymph2Cx on the NanoString platform, we have confirmed the relevance of COO determination in DLBCLs both enrolled in trials (DLCL04 of the Italian Lymphoma Foundation [FIL] ^27^ and RHDS0305 of the Italian Group for Innovative Therapies in Lymphomas [GITIL] ^28^) and retrieved from archived material (real-life). Material from patients with the same clinical characteristics (with a median age of 52 years, in stage III–IV, and with an intermediate/high to high International Prognostic Index) was profiled. As originally reported by the LLMPP, no relationship was found between IHC and GEP, and the case distribution turned out to be completely different between the two approaches. By targeted GEP, the cases with a GCB or U signature represented the clear majority and behaved significantly better than the ABC ones (more than 85% OS at five years for the former as opposed to less than 50% for the latter). Only with RHDS was the OS of ABC tumours significantly increased (75% at five years versus 25% of R-CHOP14). These results do not weaken the importance of the COO assessment. In fact, RHDS, characterised by a specific conditioning regimen (Ara-C + cisplatin) and including autologous stem cell transplantation first line, is too intense for GCB and U DLBCLs, also when considering the possible late toxicities. The latter group of tumours can benefit from the use of conventional R-CHOP.

By applying larger panels of genes, including the Pan Cancer Immune Profiling one, we observed that the expression of genes other than those related to the COO can represent additional relevant prognosticators (e.g. *BCL2* and *MYC*) in DLBCLs treated with R-CHOP/R-CHOP-like therapies. These data, which are detailed in a manuscript currently under review, fit with previous reports based on the IHC determination of BCL2 and MYC and show that the co-expression of these proteins is a strong predictor of outcome in DLBCL patients ^26, 29– 31^ (see also below in the high-grade B-cell lymphoma section).

## Microenvironment dissection

**Figure 1.**
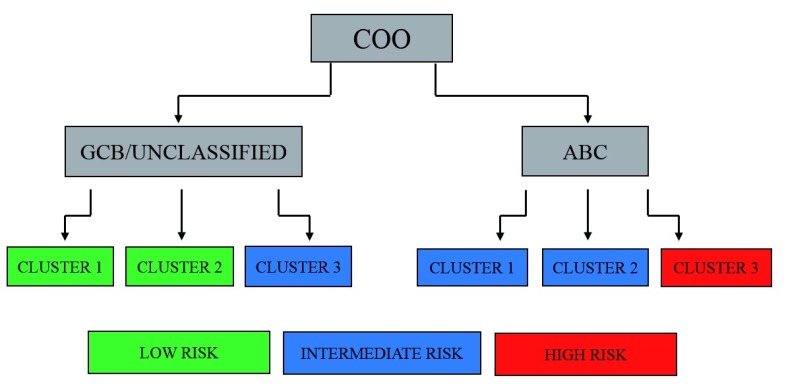
Prognostic categories of diffuse large B-cell lymphoma (DLBCL) based on cell of origin (COO) subtypes and microenvironment clusters. ABC, activated B cell-like; GCB, germinal centre B cell-like.

Besides COO determination, another important contribution to the prognosis of DLBCL is provided by the assessment of the microenvironment (ME). In 2008, Lenz and co-workers reported on two different gene signatures independent of the COO and related to the ME components, which heralded good and poor prognosis, respectively ^32^. Their study was based once again on mRNA extracted from fresh/frozen tissue and therefore found little application in daily practice, where most if not all patients have only FFPE samples available. Some attempts to substitute the results of the study based on a two-gene approach ^33^ or IHC markers remained anecdotal ^34– 36^. Recently, our group developed a 45-gene panel focusing on different ME components (mesenchymal, dendritic, and T-cell related), which is applicable to FFPE samples on the NanoString platform. It stratified the cases enrolled in the DLCL04 and RHDS0305 trials into three groups that significantly differed in terms of OS and PFS. The results were further validated by profiling real-life patients retrieved from the archive and analysing Lenz’s cases *in silico*. Finally, yet importantly, these results were reproduced on a different NanoString platform. The panel integrates the Lymph2Cx results, giving rise to a risk score that can be applied to every new case by a random forest approach ^37^ ( Figure 1). This piece of information can be of interest in light of the efficacy of some drugs (e.g. lenalidomide) on both ABC DLBCLs and ME components.

The PD1/PD-L1 axis represents a further player in the relationship between ME and neoplastic cells, which is relevant for the usage of immune checkpoint inhibitors. This issue, extensively reviewed in Xu-Monette *et al*. ^38^, is characterised by variable amounts of PD1-positive T-infiltrating lymphocytes and PD-L1-positive macrophages and/or neoplastic cells. The latter are less frequently positive in DLBCL/NOS than in primary mediastinal B-cell lymphoma, in which PD-L1 expression is frequently related to 9p24.1 alteration.

## High-grade B-cell lymphomas with and without double/triple hits

High-grade B-cell lymphomas correspond to a new provisional category of the Revised WHO Classification ^1^, which includes B-cell lymphoid neoplasms with blastoid morphology (negative for CD34, CD5, and TdT) and forms that are morphologically and/or phenotypically intermediate between DLBCL and Burkitt lymphoma. Part of these neoplasms carries double/triple rearrangements (hits) of *MYC*, *BCL2*, and/or *BCL6*. The association of *MYC* and *BCL2* rearrangements tends to be very deleterious on clinical/prognostic grounds, especially in cases where the partner gene of the former is the immunoglobulin promoter ^39– 42^. If the patient’s fitness is good, a tumour with DH/TH requires a regimen that is much more aggressive than R-CHOP ^43^. Importantly, tumours with DH/TH do not always show the above-mentioned morphologic features but can present as a conventional DLBCL/NOS; nevertheless, they are included in the new provisional category. This suggests the opportunity to perform fluorescent *in situ* hybridisation (FISH) analysis with appropriate probes in all DLBCLs or at least in the GCB forms, which are enriched in DH ^44^. Since such an approach is expensive, once again an attempt to substitute a molecular test with IHC was proposed by searching for the MYC and BCL2 products. Cases with positivity for the two proteins in more than 40% and 50% of neoplastic cells are regarded as double expressors (DE) and are thought to have a more aggressive clinical course ^26, 29– 31, 45^. However, these cut-off values have been found to have a low grade of intrapersonal and interpersonal reproducibility ^46^. In addition, no association exists between DE and DH, since the former represents about 30% of DLBCLs/NOS and the latter less than 10% ^44^. Finally, yet importantly, some DH are not DE because of gene mutations and/or the occurrence of epitopes not recognised by the anti-MYC and/or anti-BCL2 antibodies. Thus, at this time, the best compromise is to discuss with the referring clinician the usefulness of FISH analysis in each DLBCL on the basis of the patient’s general condition and the sustainability of highly aggressive therapeutic schedules.

## Next-generation sequencing

In the Revised WHO Classification, NGS studies are not regarded as mandatory for the diagnosis of malignant lymphomas. However, they are strongly recommended in light of the better understanding of the pathobiology they produce and of the practical impact they have on prognosis and ad hoc therapeutic decisions. In other words, they are thought to represent a fundamental contribution to the developing concept of precision medicine.

The term NGS applies to different techniques. Concerning DNA analysis, it can cover the whole genome (WGS), the entire exome (WES), or a series of genes selected by the investigator (targeted sequencing) based, for instance, on a statistical criterion (prevalence of a certain mutation in at least 5% of the tumours included in the study according to public databases). RNA can also undergo NGS (RNAseq); this approach is of great relevance for the detection of gene fusions caused by chromosomal translocations that cannot be shown by DNA sequencing.

During the last two years, more than 2,000 DLBCLs have been studied by NGS, more often by sequencing cases provided with fresh or frozen tissue, which allows the extraction of optimally preserved DNA ^47– 50^. In this respect, one should remember that formalin fixation (which should never exceed the 24-hour limit) and paraffin embedding cause variable degrees of DNA degradation that may hamper the interpretation of results. In our experience, successful targeted sequencing in more than 50% of FFPE samples can be achieved by extracting DNA using the Covaris technology.

**Figure 2.**
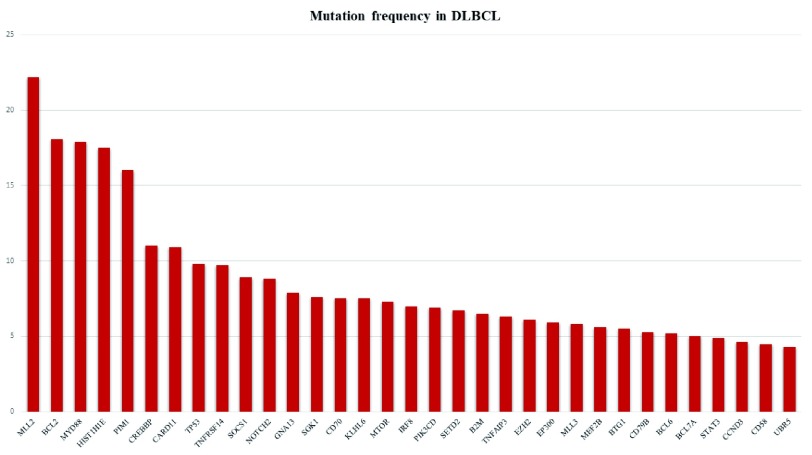
Most frequently observed gene mutations in diffuse large B-cell lymphoma (DLBCL) according to personal experience. Most frequently observed gene mutations in diffuse large B-cell lymphoma (DLBCL) according to personal experience.

Based on the results of the above-mentioned studies, the most frequent recurrent mutations affect the following genes: *MLL2, BCL2, MYD88, HIST1H1E, PIM1, CREBBP, CARD11, TP53, TNFRSF14, SOCS1, NOTCH2, GNA13, SGK1, CD70, KLHL6, MTOR, IRF8, PIK3CD, SETD2, B2M, TNFAIP3, EZH2, EP300, MLL3, MEF2B, BTG1, CD79B, BCL6, BCL7A, STAT3, CCND3, CD58*, and *UBR5*. Importantly, some of them are provided with prognostic impact. This is the case for *TP53* mutations, with special reference to those occurring in the DNA-binding domain ^51^. In addition, many mutations do not occur at random but are related to the COO ^52^ ( Figure 2) and possibly to the ME ^50^. The knowledge of the mutational landscape along with the COO, the ME, and cytogenetics represents pivotal information for the use of tailored therapies. In fact, genetic aberrations can cause the synthesis of anomalous proteins that may be targeted by biological agents (e.g. venetoclax and histone deacetylase inhibitors) or the deregulation of pathways at different levels, which may be restored by specific drugs (e.g. ibrutinib). The appropriate prescription of the new drugs based on molecular data will not only enhance their success rate by avoiding unwanted toxicity without efficacy in patients lacking the target but also contribute to limiting public assistance expenditure ^53^.

A further application of NGS studies is the so-called liquid biopsy, which is currently a research tool in light of the costs required by ultra-deep sequencing. It is hoped that these costs will decrease soon, making the approach applicable to most lymphoma patients. The liquid biopsy will never replace the diagnostic biopsy, but it effectively integrates the latter with minimal burden for the patient. In fact, tumour circulating free DNA (cfDNA) is captured from a sample of venous peripheral blood. Its sequencing with a coverage of 5,000X to 7,000X has allowed us to observe that the mutational landscape of DLBCL can be broader than the one shown by the diagnostic biopsy, since different mutations can occur at the different anatomic sites associated with the tumour ^54, 55^. This finding has changed the concept that a malignant lymphoma is a systemic homogeneous disease irrespective of the site of involvement: a certain degree of heterogeneity does occur, which may be related to subclones and/or ME influence. In addition, the liquid biopsy represents a very effective tool for monitoring the response to therapy and minimal residual disease. In fact, the clearance of the mutational landscape in cfDNA indicates complete molecular remission. In contrast, the persistence of detectable mutations and/or the appearance of new aberrations herald a lack of response to therapy or early disease relapse ^54, 55^.

## Future perspectives

**Figure 3.**
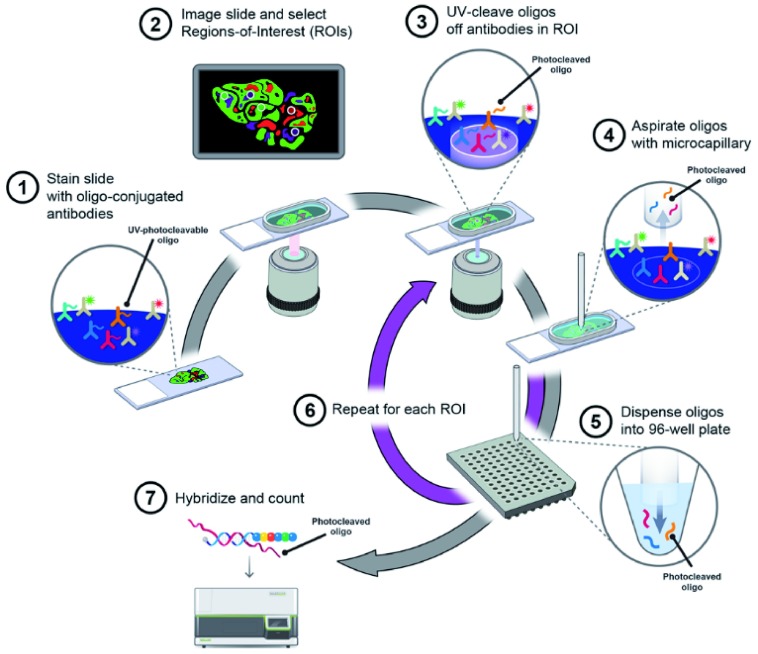
Schematic representation of the digital spatial profiling system (from NanoString documentation). Schematic representation of the digital spatial profiling system.

A new platform has recently been developed by NanoString pointing to digital spatial profiling: it will become available by the beginning of 2019. This device will possibly allow the selection of the different components of each tumour under microscopic control and by the usage of a panel of 40 markers uncovered by immunofluorescence multiplex techniques. Accordingly, one might dissect the ME from the tumour environment as well to look for the heterogeneity of the latter by selectively extracting mRNA and DNA ( Figure 3). The goal is to refine the interpretation of the pathobiology of malignant lymphomas and the prediction of sensitivity to targeted drugs.

## Abbreviations

ABC, activated B cell-like; cfDNA, circulating free DNA; COO, cell of origin; DE, double expressors; DH/TH, double hits/triple hits; DLBCL, diffuse large B-cell lymphoma; FFPE, formalin-fixation, paraffin-embedding; FISH, fluorescent *in situ* hybridisation; GCB, germinal centre B cell-like; GEP, gene expression profiling; IHC, immunohistochemistry; LLMPP, Lymphoma/Leukemia Molecular Profiling Project; ME, microenvironment; NGS, next-generation sequencing; NOS, not otherwise specified; OS, overall survival; PFS, progression-free survival; U, undetermined; WHO, World Health Organization.
